# Draft genome sequence data of a chromium reducing bacterium, *Bacillus licheniformis* strain KNP

**DOI:** 10.1016/j.dib.2020.106640

**Published:** 2020-12-15

**Authors:** Pankaj Kumar Arora, Rupali Mishra, Rishabh Anand Omar, Raj Shekhar Saroj, Alok Srivastava, Sanjay Kumar Garg, Vijay Pal Singh

**Affiliations:** a Department of Microbiology, Babasaheb Bhimrao Ambedkar University, Lucknow 226025, India; b Department of Plant Science, Faculty of Applied Sciences, MJP Rohilkhand University, Bareilly, India

**Keywords:** Chromium, *Bacillus*, Chromate transporter, Reduction

## Abstract

A chromium-reducing bacterium designated as strain KNP was isolated from a sample collected from a tannery effluent of Kanpur, India. Phylogenetic analysis based on the 16S rRNA gene sequences revealed that strain KNP belonged to the *Bacillus* genus and showed 100% similarity with *Bacillus licheniformis*. Furthermore, average nucleotide identity and digital DNA-DNA hybridization between strain KNP and its closely related strains confirmed its affiliation with *Bacillus licheniformis* species*.* Whole-genome sequencing of *Bacillus licheniformis* KNP was performed using the Illumina Hiseq platform. Here, we present the draft genome sequence of *Bacillus licheniformis* KNP. The total size of the draft assembly was 4,280,093 bp, distributed into 21 contigs with an N50 value of 4,186,229. The genome has 45.9% G + C content, 4255 coding sequences and 86 putative RNA genes. This Whole Genome Shotgun project has been deposited at DDBJ/ENA/GenBank under the accession JACDXS000000000. The version described in this paper is version JACDXS010000000.

## Specifications Table

**Table:** 

Subject	Biochemistry, Genetics and Molecular Biology (General)
Specific subject area	Genomics and Microbiology
Type of data	Draft genome sequence data in FASTA format, table and figures
How data were acquired	Whole genome sequence of *Bacillus licheniformis* KNP was sequenced with Illumina HiSeq system.
Data format	Raw, analyzed and assembled genome sequences
Parameters for data collection	Strain KNP reduced hexavalent chromium up to concentrations of 1000 ppm. Genomic DNA was isolated from a pure culture of *Bacillus licheniformis* KNP.
Description of data collection	Whole-genome sequencing, assembly, and annotation
Data source location	*Bacillus licheniformis* KNP was isolated from a tannery effluent sample collected from Kanpur (26.4670°N 80.3500°E), India.
Data accessibility	Data is publicly available at NCBI Genbank from the following links: https://www.ncbi.nlm.nih.gov/nuccore/JACDXS000000000 https://www.ncbi.nlm.nih.gov/bioproject/PRJNA645298 https://www.ncbi.nlm.nih.gov/biosample/SAMN15501749

## Value of the Data

• Whole Genome sequence of *Bacillus licheniformis* strain KNP could provide valuable information about chromium resistance and its transformation.
• The data of this article could be useful for scientists and students working in the field of environmental microbiology, environmental biotechnology, genomics and genetic engineering.
• This genome data could be valuable resource for comparative genomic analysis among *Bacillus licheniformis* strains.
• Based on genome data, *Bacillus licheniformis* strain KPN could be a potential strain for study of heavy metal stress, bacterial chemotaxis, and various enzymes production.

## 1 Data Description

**Fig. 1:**
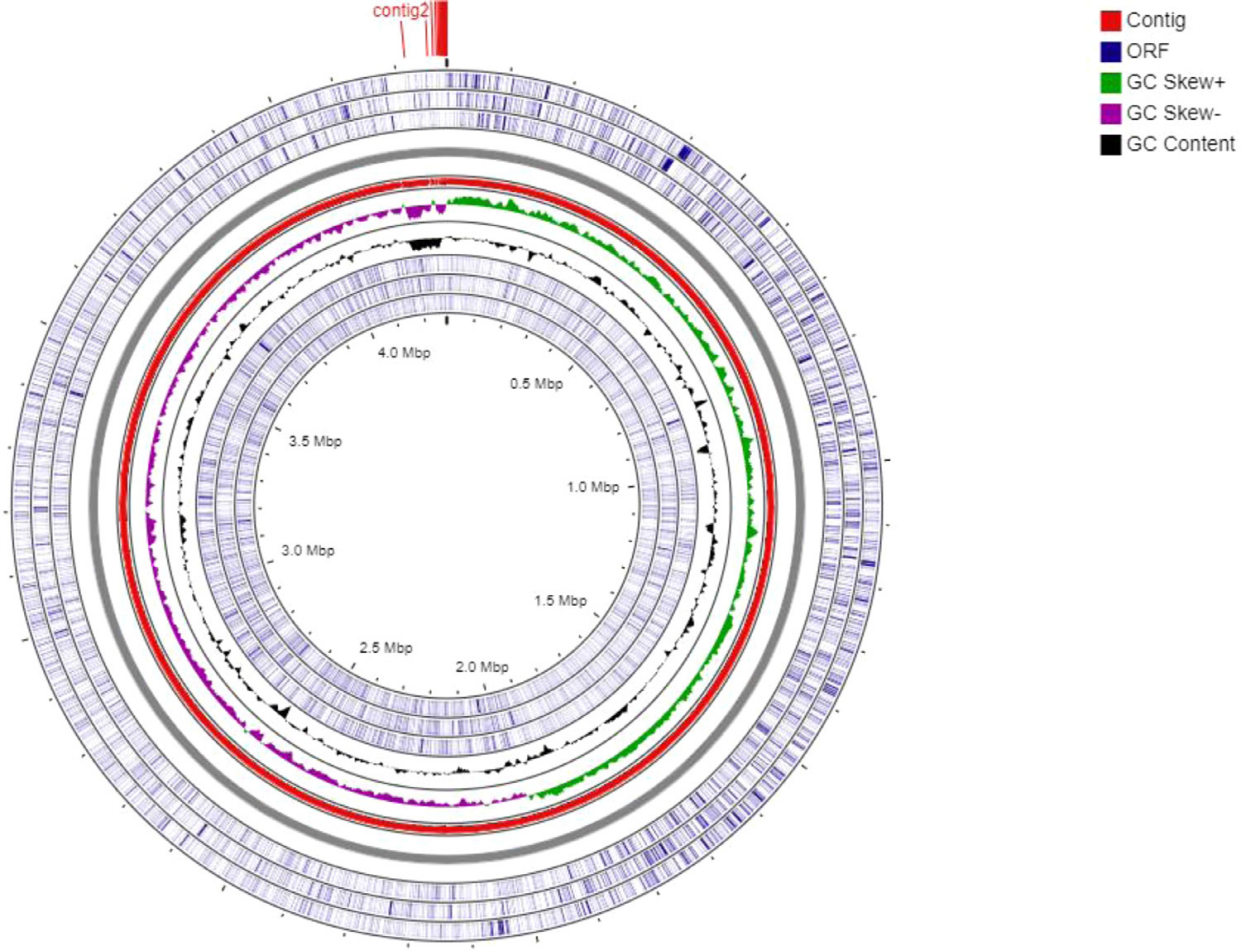
Circular map of *Bacillus licheniformis* strain KNP. The following rings were included: i) ORFs (blue color); (ii) Contigs (red color); iii) Positive GC Skew (green color); (iv) Negative GC Skew (violet color); (v) G + C content (black color). (For interpretation of the references to color in this figure legend, the reader is referred to the web version of this article.)

*Bacillus licheniformis* KNP was isolated from a tannery effluent sample collected from Kanpur, India. Strain KNP reduced hexavalent chromium completely within 48 h when it was grown in nutrient media containing 1000 mg/L potassium dichromate under shaking conditions (200 rpm). The genome features of strain KPN were summarized in Table 1. The assembled genome of *Bacillus licheniformis* KNP comprised 21 contigs with a total size of 4,280,093 bp and N50 value of 4,186,229. The genome G + C content was 45.9%. Based on the genome annotation, a total of 4434 genes were predicted in which, 4255 of them were responsible for coding specific proteins while 86 and 93 of them were coded for RNA genes (77 tRNAs, 5 ncRNAs, 4 16S-23S-5S rRNAs) and pseudogenes, respectively. A circular map of genome of *Bacillus licheniformis* KNP was represented in Fig. 1.

**Table 1:** Genome features of *Bacillus licheniformis* strain KNP.

Features	Value	Percentage
Number of contigs	21	100
Genome size (bp)	4,280,093 bp	100
G + C	1,964,563 bp	45.9
Genes (total)	4434	100
Protein coding genes	4255	95.96
RNA genes	86	1.94
5S rRNA gene	2	0.05
16S rRNA gene	1	0.02
23S rRNA gene	1	0.02
tRNAs	77	1.74
ncRNAs	5	0.11
Pseudo Genes (total)	93	2.10

**Fig. 2:**
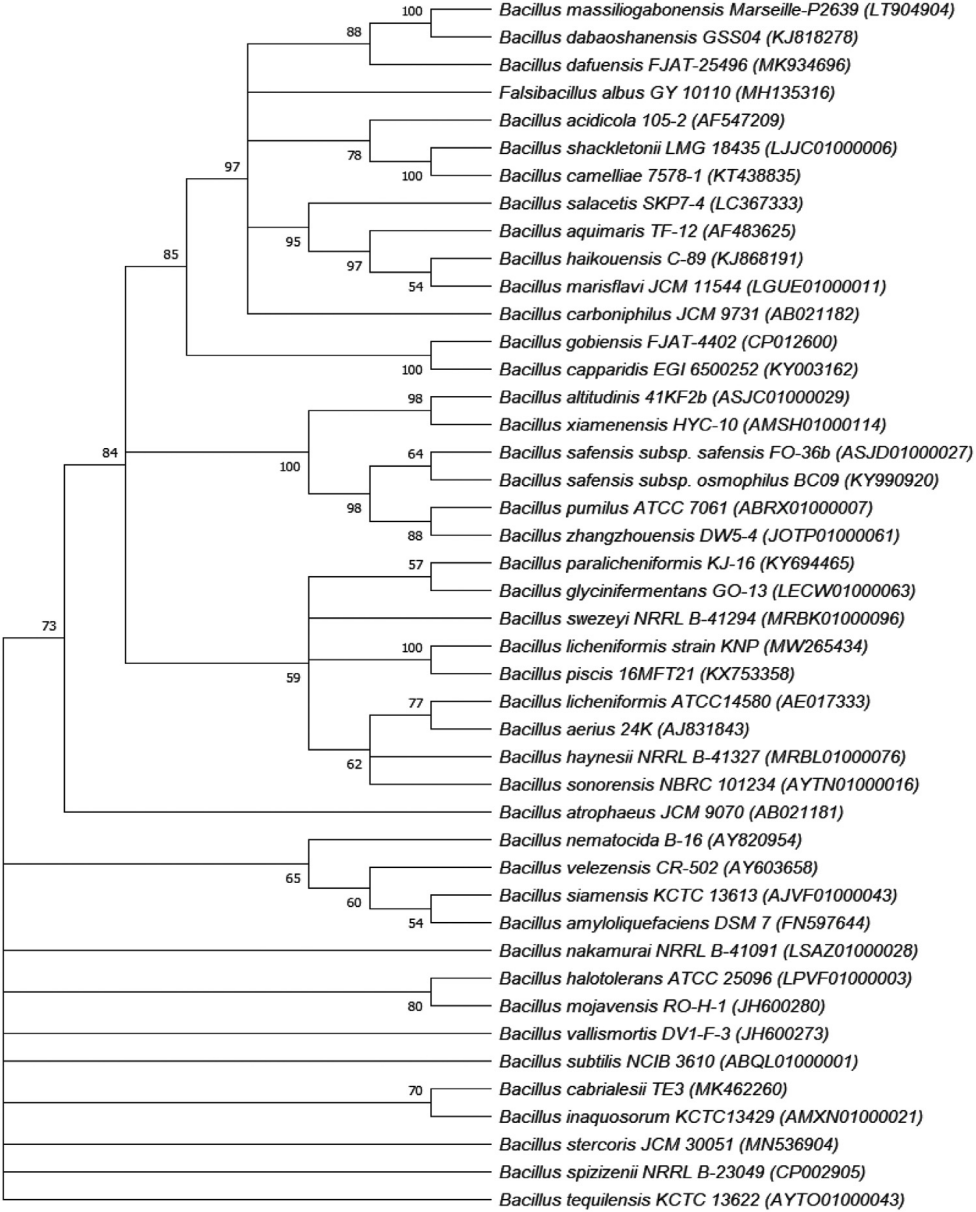
Neighbor-joining tree based on the16S rRNA gene sequence (1325 nt) showing the relationships among strain KNP and its related type strains The numbers at branch nodes indicate bootstrap percentages derived from 1000 replications; only values >50 % are shown.

The 16S rRNA gene sequence of strain KNP was deposited to NCBI Genbank under accession number MW265434. Phylogenetic analysis based on the 16S rRNA gene sequences revealed that strain KNP was affiliated to *Bacillus* (Fig. 2) and exhibited 100% 16S rRNA gene similarity with *Bacillus licheniformis* ATCC 14580*.* Furthermore, genome-based taxonomic analysis showed that strain KNP exhibited high average nucleotide identity (ANI) value (99.54%) as well as high digital DNA-DNA hybridization (dDDH) value (96.50%) with *Bacillus licheniformis* (Table 2). Based on the cutoff values on species delimitation established for ANI (> 95–96%) [1] and dDDH value (> 70%) [2], strain KNP was strongly affiliated to *Bacillus licheniformis.*

**Table 2:** 16S rRNA gene similarity (> 98.7%), ANI and dDDH values of strain KNP with its closely related species.

Closely related Species	16S rRNA sequence similarity	OrthoANI value (%)	dDDH value (%)
*Bacillus licheniformis* ATCC 14,580	100.00%	99.54%	96.50%
*Bacillus paralicheniformis* KJ-16	99.92%	94.65%	57.70%
*Bacillus haynesii* NRRL B-41,327	99.92%	95.65%	64.10%
*Bacillus sonorensis* NBRC 101,234	99.84%	81.82%	24.40%
*Bacillus glycinifermentans* GO-13	99.84%	80.93%	23.60%
*Bacillus swezeyi* NRRL B-41,294	99.45%	83.38%	26. 20%
*Bacillus subtilis* NCIB 3610	98.90%	72.53%	18.50%
*Bacillus cabrialesii* TE3	98.90%	72.73%	18.30%
*Bacillus inaquosorum* KCTC 13,429	98.90%	72.90%	18.60%
*Bacillus stercoris* D7XPN1	98.90%	72.60%	18.50%
*Bacillus spizizenii* TU-B-10	98.90%	72.80%	19.10%
*Bacillus tequilensis* KCTC 13,622	98.82%	72.83%	18.10%
*Bacillus siamensis* KCTC 13,613	98.82%	72.69%	18.40%
*Bacillus amyloliquefaciens* DSM 7	98.74%	72.49%	19.20%
*Bacillus atrophaeus* NRRL NRS 213	98.74%	72.68%	18.10%
*Bacillus nakamurai* NRRL B-41,091	98.74%	72.59%	18.40%
*Bacillus halotolerans* FJAT-2398	98.74%	72.61%	18.40%
*Bacillus velezensis* NRRL B-41,580	98.68%	72.67%	18.40%
*Bacillus mojavensis* KCTC 3706	98.67%	72.91%	18.30%

Functional annotation of genome of strain KPN revealed presence of three chromate transporters that involve in chromium resistance through chromate efflux mechanism [3]. Furthermore, putative genes related to the chromium reduction such as nitroreductase [4], quinone reductase [5], and azoreducatase [6] were also identified in the genome of strain KNP. In addition, genes coding the proteins involved in arsenic resistance and reduction [7] such as arsenate reductase (thioredoxin), arsenical efflux pump membrane protein ArsB, arsenical pump-driving ATPase, arsenate reductase family protein, arsenite efflux transporter metallochaperone ArsD, arsenic transporter were also identified in the KNP genome. Several other genes involved in heavy metal resistance including heavy metal translocating P-type ATPase, divalent metal cation transporter, Nramp family divalent metal transporter, nickel ABC transporter, nickel/metallophore periplasmic binding protein, metal ABC transporter ATP-binding protein, metal ABC transporter permease, metal ABC transporter substrate-binding protein, MerR family transcriptional regulator, copper-sensing transcriptional repressor CsoR, BlaI/MecI/CopY family transcriptional regulator, TetR/AcrR family transcriptional regulator, MarR family transcriptional regulator, GbsR/MarR family transcriptional regulator, LysR family transcriptional regulator, Transcriptional regulator MntR, Zn(II)-responsive metalloregulatory transcriptional repressor CzrA, MgtC/SapB family protein, Spx/MgsR family RNA polymerase-binding regulatory protein were also detected in the KNP genome. Moreover, genes related to bacterial chemotaxis were also identified in the KNP genome.

PHAST analysis [8] was performed to identify prophages in the genome of strain KNP. Total 5 prophage regions were identified, of which 3 regions were intact, 2 regions were incomplete. Intact regions of prophages were located between positions 124,430–169,526, 1,312,385–1,348,631 and 3,119,232–3,183,647 bp, respectively.

To predict the potential of *Bacillus licheniformis* KNP to produce secondary metabolites, the genome of this strain was analyzed with antiSMASH server v.5.0 [9]. The results showed that the genome contained eleven gene clusters coding for enzymes involved in the biosynthesis of bacteriocins, nonribosomal peptides, thiopeptide, siderophores, betalactone, terpenes, lanthipeptides, type III polyketides, lassopeptides, and tRNA-dependent cyclodipeptide. Two of the non-ribosomal peptide synthetase (NRPS) gene clusters exhibited 100% and 53% similarity with the lichenysin and bacillibactin gene clusters, respectively. Moreover, lanthipeptide and betalactone gene clusters showed 100% and 53% similarity with lichenicidin VK21A1/VK21A2 and fengycin gene clusters, respectively. Based on genome analysis, strain KPN has a potential strain for study of heavy metal stress, bacterial chemotaxis, and various enzymes production.

## 2 Experimental Design, Materials and Methods

### 2.1 Collection of sample and isolation of chromium resistance bacteria

*Bacillus licheniformis* KNP was isolated from a tannery effluent sample collected from Kanpur (26.4670°N 80.3500°E), India. Briefly, a tannery effluent sample was collected in screw capped sterilized bottle from Kanpur, India. For isolation of hexavalent chromium resistance bacteria, 1 ml of water sample was inoculated in 500 ml Nutrient broth and 500 ppm potassium dichromate for 72 h. The sample was serially diluted and poured into nutrient agar plate containing 500 ppm potassium dichromate and plates were inoculated at 30 °C for 48 h. A total eighteen morphotypes were selected and purified and preserved at −10 glycerol vials.

### 2.2 Hexavalent chromium transformation assay

All eighteen bacteria were screened for the reduction of hexavalent chromium at various concentrations (200–1200 ppm) of potassium dichromate by the diphenylcarbazide colorimetric method [10]. Out of eighteen bacteria, only one bacterium designated strain KNP showed the hexavalent chromium reduction up to concentrations of 1000 ppm.

### 2.3 Bacterial cultivation and DNA isolation

*Bacillus licheniformis* KNP was cultivated on nutrient agar plate at 37 °C. A single colony of strain KNP was grown overnight in Luria-Bertani media under shaking conditions. The culture was centrifuged and pellet was used for DNA extraction. Genomic DNA was extracted using the DNAminikit (Qiagen, Germantown, MD, USA), according to the manufacturer's instruction.

### 2.4 Genome sequencing, assembly, and annotation

A whole-genome sequencing library was constructed using the Nextera XT DNA library preparation kit, according to the manufacturer's instruction. The libraries were sequenced using the Hiseq platform (Illumina, San Diego, CA, USA), with 150-bp paired-end reads and 1900-fold genome coverage. FastQC was used to monitor the initial quality of the raw sequencing data [11]. The raw reads and adapter contam-inations were trimmed with Trim galore 0.6.5 [12] and primary assembly was performed using Unicycler version v0.4.8 [13]. Default parameters were used for all software unless otherwise specified. Annotation was performed using NCBI Prokaryotic Genome Automatic Annotation pipeline (PGAAP) [14]. The genome was analyzed with the PHAge Search Tool (PHAST) to identify prophages [8]. The potential secondary metabolite biosynthetic gene clusters (BGCs) were identified in the genome using antiSMASH v5.0 [9]. The graphical circular map of the complete genome was constructed and visualized using CGView Server [15].

### 2.5 16S rRNA gene sequence and phylogenetic analysis

The 16S rRNA gene sequence of strain KNP (1325 nt) was retrieved from the draft genome sequence of strain KNP with RNAmmer [16]. The 16S rRNA gene sequence of strain KNP was analyzed by EzBiocloud to determinate its more closely related strains [17]. The 16S rRNA gene sequences of all closely related species were retrieved from EzBiocloud database. All sequences were aligned with ClustalW [18] and phylogenetic tree was constructed by the neighbour joining method with MEGA X software package [19].

### 2.6 Average nucleotide identity and digital DNA-DNA hybridization

Average nucleotide identity (ANI) between genomes of strain KNP and closely related species were determined by the OrthoANI algorithm [20] and digital DNA-DNA hybridization (dDDH) values were determined using genome-to-genome distance calculator (GGDC) 2.1 by BLAST [21].

## Data Availability

This Whole Genome Shotgun project has been deposited at DDBJ/ENA/GenBank under the accession JACDXS000000000. The version described in this paper is version JACDXS010000000.

## Declaration of Competing Interest

The authors declare that they have no known competing financial interests or personal relationships that could have appeared to influence the work reported in this paper.
